# CRISPR/Cas9-Mediated Targeted DNA Integration: Rearrangements at the Junction of Plant and Plasmid DNA

**DOI:** 10.3390/ijms23158636

**Published:** 2022-08-03

**Authors:** Natalya V. Permyakova, Tatyana V. Marenkova, Pavel A. Belavin, Alla A. Zagorskaya, Yuriy V. Sidorchuk, Elena V. Deineko

**Affiliations:** Federal Research Center Institute of Cytology and Genetics, Siberian Branch of Russian Academy of Sciences, pr. Lavrentieva 10, Novosibirsk 630090, Russia; marenkova@bionet.nsc.ru (T.V.M.); belavin@bionet.nsc.ru (P.A.B.); zagorska@bionet.nsc.ru (A.A.Z.); sidorch@bionet.nsc.ru (Y.V.S.); deineko@bionet.nsc.ru (E.V.D.)

**Keywords:** Arabidopsis, gene editing, knock-in, HDR, NHEJ

## Abstract

Targeted DNA integration into known locations in the genome has potential advantages over the random insertional events typically achieved using conventional means of genetic modification. We studied the presence and extent of DNA rearrangements at the junction of plant and transgenic DNA in five lines of *Arabidopsis thaliana* suspension cells carrying a site-specific integration of target genes. Two types of templates were used to obtain knock-ins, differing in the presence or absence of flanking DNA homologous to the target site in the genome. For the targeted insertion, we selected the region of the histone H3.3 gene with a very high constitutive level of expression. Our studies showed that all five obtained knock-in cell lines have rearrangements at the borders of the integrated sequence. Significant rearrangements, about 100 or more bp from the side of the right flank, were found in all five plant lines. Reorganizations from the left flank at more than 17 bp were found in three out of five lines. The fact that rearrangements were detected for both variants of the knock-in template (with and without flanks) indicates that the presence of flanks does not affect the occurrence of mutations.

## 1. Introduction

The development and improvement in methods of molecular biology and genetic engineering, as well as the rapid development of genomic editing methods using CRISPR/Cas9, allows researchers to set themselves the task of targeted delivery of target genes to almost any selected constitutively transcribed genome regions. This will make it possible in the future to abandon the laborious screening of a large number of transgenic lines and directly obtain highly productive lines producing recombinant proteins with target genes delivered to transcriptionally active regions. Site-specific endonucleases, the best known of which is the Cas9 endonuclease, are capable of producing DNA double-strand breaks (DSBs) in a given region of the genome, which can then be repaired by one of two main eukaryotic mechanisms: non-homologous end joining (NHEJ) or homologous directed repair (HDR). In all eukaryotes, with the exception of yeast, repair mainly occurs according to one of the varieties of the NHEJ mechanism [1,2,3]. When the DSB generates overhangs, NHEJ can mediate the targeted introduction of a double-stranded DNA template with compatible overhangs [4,5]. When a template with regions of homology to the sequence surrounding the DSB is available, the DNA damage can be repaired by HDR, and this mechanism can be exploited to achieve precise gene modifications or gene insertions [6,7,8].

The study of regions of integration of DNA inserts (knock-ins) using CRISPR/Cas9 technology is of great interest from the point of view of revealing the mechanisms of integration of transgenic DNA into the plant genome and establishing the features of the functioning of cell repair systems. The integration of the delivered fragment into the region of the DNA DSB, carried out by the Cas9 nuclease, occurs using the same repair mechanisms as in the integration of exogenous DNA fragments during modification of the plant genome using genetic engineering methods. It was established that in the case of knockout genes encoding key repair proteins by the NHEJ mechanism, the integration of exogenous DNA (T-DNA) occurred due to an alternative mechanism (microhomology-mediated end joining, MMEJ) [9,10]. According to Kleinboelting et al., additional knockouts of the genes of the components of the MRX complex, which provides repair by the MMEJ mechanism, in the *Arabidopsis thaliana* line with knockouts of the NHEJ components should lead to the emergence of plants resistant to T-DNA integration [11]. A wide range of mutations obtained by integrating exogenous DNA fragments into the *A. thaliana* plant genome during genetic transformation [12] and currently represented by extensive collections in the data bank (GABI-Kat) attracts the close attention of researchers who use bioinformatics methods to analyze insertion integration regions [13,14]. The study of changes in the nucleotide composition in regions flanking the insertion of the target gene delivered to the target region using the CRISPR/Cas9 system can provide new information about the functioning of repair systems in plants. In this regard, the accumulation of new data on rearrangements in target gene delivery regions during genome editing is of great interest and is the subject of our future research.

Most of the genetic constructs intended for the delivery of transgenes to the target region using the CRISPR/Cas9 genomic editing technology include flanking DNA regions homologous to the insertion site [15,16,17,18]. To increase the efficiency of the delivery of the target construct to a given region of the genome of *A. thaliana* plants [15] and *Zea mays* [18], a technique is used that allows the release of the target fragment in the cell in the form of a linear template from the circular plasmid DNA with the help of Cas9 nuclease. Using this approach, a number of *A. thaliana* suspension cell lines carrying knock-ins in the region of the histone H3.3 gene were created. The template for knock-in carried two genes—A *dIFN* gene encoding deltaferon (dIFN) as the target gene and a *nptII* gene giving the resistance to kanamycin as a marker. After a series of biolistic transformations, three lines were obtained using a knock-in template carrying flanking DNA homologous to the insertion site in the plant genome and presumably integrated into the genome by the HDR mechanism. Two lines were obtained using a knock-in template without flanking DNA and presumably integrated into the genome by the NHEJ mechanism [19].

The aim of our work was to study five *A. thaliana* suspension cell lines carrying site-specifically integrated target genes in the region of the histone H3.3 gene and to show the presence or absence of mutations at the junction of plant and transgenic DNA as well as to evaluate the differences in frequency and extent of the mutation arising during insertion through the mechanisms of HDR and NHEJ.

## 2. Results

### 2.1. Delivery of the Genetic Constructs to the Selected Target Region of the A. thaliana Genome

After 10 biolistic transformations, we obtained 15 kanamycin resistant calli with construct pIFN (H3.3).2 and 20 kanamycin resistant calli with construct pIFN (H3.3).3. To confirm the integration of the target gene into the target region of the histone H3.3 gene, PCR was used with primers, one of which was located inside the transferred construct, and the other on the plant DNA region outside the flanking sequence present in the construct (Lo_plan3 and Up_H3.3_1, Table 1). Alignment of the nucleotide sequence of this PCR fragments showed identity with *A. thaliana* histone superfamily protein (AT4G40040) from one side and identity with vector DNA (namely, with the promoter of the *nptII* gene) from the other side. Additionally, control PCR was performed with primers located inside the construct (Lo_plan3 and Up_H3.3, dIFN1 and dIFN2) (Table 1).

Not all kanamycin-resistant lines turned out to be knock-ins; most of the resulting lines were the result of random insertion of the target construct into the Arabidopsis genome. Two genetic constructs differed in the delivery efficiency of target genes in a target region. After 10 biolistic transformations, the largest number of knock-ins in the target site, six lines, were obtained using the pIFN (H3.3).3 genetic construct, while only three were obtained using the pIFN (H3.3).2 construct. The production of lines with knock-in is described in more detail in our previous work [19].

**Figure 1:**
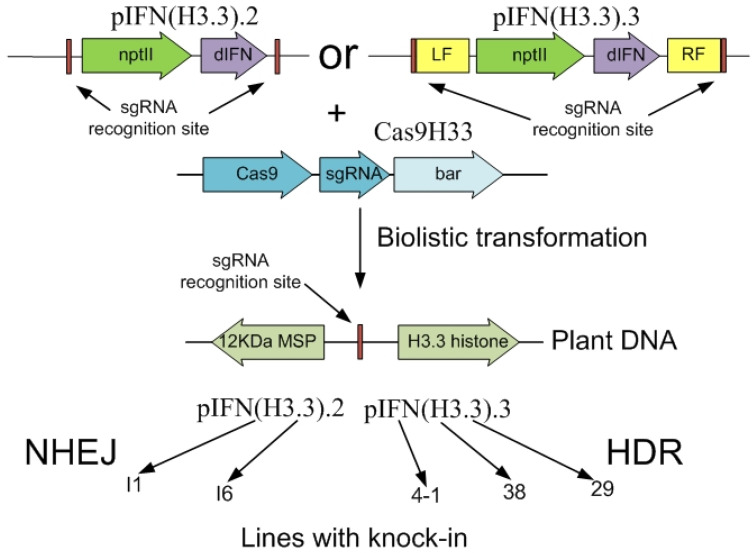
The general scheme for obtaining lines with knock-in. Designations: nptII, neomycin phosphotransferase II gene providing plant cell resistance to kanamycin; dIFN, DNA sequence encoding the target dIFN protein; LF and RF, left and right flanking sequences homologous to the corresponding regions in the *A. thaliana* genome; sgRNA—single guide RNA gene; Cas9—endonuclease Cas9 gene; bar—phosphinothricin resistance gene; 12 KDa MSP—12 kDa subunit of microsomal signal peptidase (At4g40042) gene of *A. thaliana*; H3.3 histone—histone H3.3 gene HTR5 (At4g40040) of *A. thaliana*; NHEJ—non-homologous end joining; HDR—homologous directed repair.

After biolistic transformation, calli cultivated on a culture medium with kanamycin represent a heterogeneous mass of cells, including both cells with knock-in events in the target region and cells with the integration of target genes into random regions of the genome. Unfortunately, the procedures for the analysis and cultivation of callus, which involve the separation of heterogeneous material into parts, led to the loss of part of the knock-ins identified at the early stages of selection. Thus, as a result, 5 stable monoclonal lines of suspension cell cultures with knock-in were finally obtained: two (I1 and I6) using the pIFN (H3.3).2 genetic construct, which was inserted into the plant genome presumably by the NHEJ mechanism, and three (29, 38 and 4-1) using the pIFN (H3.3).3 construct, its insertion into plant genome, presumably occurred by the HDR mechanism, due to the presence in the construct of flanking sequences homologous to the insertion site. The general scheme for obtaining lines with knock-in into the target region of the histone H3.3 gene of *A. thaliana* is shown in Figure 1.

### 2.2. Sequencing of the Junction of Plant and Transgenic DNA

The results for the analysis of the alignment of fragments characterizing the right and left junctions of plant and transgenic DNA for all five obtained *A. thaliana* suspension cell lines with knock-ins are presented in Table 2.

#### 2.2.1. Analysis of Line 29

A total of 10 fragments were obtained for the right flank (RF) and 2 fragments for the left flank (LF). Multiple alignment of the fragments adjacent to the RF revealed small areas of homology with the GST tag sequence located in front of the RF. Two isolated fragments, when aligned with the sequences of the constructs used to obtain knock-ins, also revealed the insertion of two P-NOS promoter regions in a row, 98 bp long in forward and 69 bp long in reverse orientation, between the GST tag and plant DNA, immediately followed by the sequence of flanking plant DNA without the first 4 bp. At the LF, insertions of parts of the template for knock-in were also observed, immediately after the end of the sequence of the LF, there were 34 bp that do not have homology in the database, then there was an 83 bp sequence homologous to the DNA region before the T-NOS terminator (part of the *nptII* gene), then came the P-NOS sequence of the *nptII* gene promoter, which should be located in this place, but in line 29 this promoter lacked the first 46 bp.

#### 2.2.2. Analysis of Line 38

Most of the sequenced fragments (five fragments) in the search for homology in BLAST showed a match with the sequences of the *A. thaliana* genome, but not directly related to our target site in genome. There was also one fragment that showed homology in the left half of the fragment with the plant DNA sequence immediately preceding the LF, and in the right half with the RF sequence immediately following the insertion site. Two more sequenced fragments showed homology with a region from the middle of the GST gene sequence lying immediately near the RF, and then with the pIFN (H3.3).3 plasmid sequence lying immediately after the RF sequence, in sections of 150–160 bp in forward and reverse orientation and interspersed with smaller sections of plasmid DNA of unknown origin, also forming hairpins. Alignment of the sequence obtained for the LF of the construct showed minimal mutation at the insertion site, namely, the presence of two additional cytosine residues at the junction of the *A. thaliana* genomic DNA and the DNA of the target insertion.

#### 2.2.3. Analysis of Line 4-1

In total, seven DNA fragments were obtained for the RF of the knock-in in line 4-1. Two homologous fragments did not align either with the insertion site or with the plasmids used to create knock-ins, and when searching for homology in BLAST, they showed a match with the sequences of the *A. thaliana* genome, but not directly related to our insertion site. The other two fragments were also homologous to each other and had homology with a region from the pIFN (H3.3).3 plasmid used to create knock-ins, located in the plasmid near the LF, outside the sequence of the knock-in template. Alignment of the sequence obtained for the LF of the knock-in showed the insertion of a small additional sequence of 24 bp, which, when searched for homology in the database using BLAST, showed homology with a region of the 4 th chromosome of *A. thaliana*. The same insertion occurred at the junction of plant and plasmid DNA in line I1. The sequence of plant DNA from the LF side was completely preserved.

#### 2.2.4. Analysis of Line I1

In the study of line I1, sequence alignment of seven PCR fragments for the RF, obtained by various methods, made it possible to conclude that there were large rearrangements in the region of integration of the right side of our template into the plant genome. Rearrangements began 15 nucleotides from the end of the GST sequence, i.e., from the end of our construct; alignment with the sequences of the plasmids used to create the knock-in showed that after the target knock-in sequence, the insertion of 400 nucleotides also occurred, which had homology with the sequence of the Cas9 gene from the Cas9H33 plasmid. The methods we used did not allow us to find the beginning of the plant DNA sequence and determine the exact length of the rearrangement. Alignment of the sequences obtained for the LF of the insert for line I1 showed the presence of an insertion of 17 bp identical to the insert at the left border for line 4-1. At the same time, it should be noted that the insertion in these two lines presumably occurred according to different mechanisms—4-1 by the HDR mechanism, I1 by the NHEJ mechanism. No other rearrangements were found in plant or transgenic DNA at the LF.

#### 2.2.5. Analysis of Line I6

For line I6, sequence alignment of 6 PCR fragments for the RF with the DNA sequence of our insert revealed the presence of an insertion of 2 nucleotides (AC) after the end of the GST gene sequence. At the same time, instead of plant DNA, after the end of the insertion, there were three insertions that showed homology with different regions of the pIFN (H3.3).2 and Cas9H33 plasmids—170 bp, 260 bp in forward and reverse orientation. The fragments were homologous to the regions that match in both plasmid vectors that were used in this study, so it was not possible to correctly determine the source of insertion of these fragments into plant DNA. We failed to identify the beginning of plant DNA. Alignment of the sequences obtained for the LF of the construct showed minimal disturbances at the insertion site—the presence of two additional cytosine residues at the junction of the genomic DNA of *A. thaliana* and the DNA of the transgenic insertion, as well as in line 38.

## 3. Discussion

Site-specific gene integration via CRISPR/Cas9 relies on endogenous DNA repair machinery, which is used to repair genomic DNA damage due to DSBs created by Cas9 [6]. DSBs activate the cell’s DNA repair machinery making the break site accessible to a donor template for potential GT [3,20,21]. Due to the flexibility and ease of the design, CRISPR-Cas9 has become an important tool for generating targeted DSBs for genome-engineering applications [22,23,24,25]. In somatic cells, NHEJ and MMEJ, which mainly involves ligation of unrelated sequences or sequences with micro-homologies, are the primary DNA repair pathways [26]. While targeted mutagenesis using CRISPR-Cas9 is now routine in plants that are amenable for transformation, HR-mediated targeted insertion or allele replacement remains a challenge [7,27].

In a natural system, the template used in the repair process via HDR is the sister chromatid of the corresponding damaged region. This repair mechanism is less prone to error than NHEJ since it uses an identical unbroken DNA sequence as a template for the repair [26]. However, as long as a sequence is homologous to the regions that flank the DNA cleavage point, any sequence could be used as a template to resynthesize the DSB. Gene targeting explores this characteristic using an exogenous sequence, instead of the sister chromatid, as a template, leading the cell to introduce the genomic modification of interest via HDR [28,29]. Genome editing by HDR has been used to promote insertions into the genome and to exchange certain bases or regions in the target sequence [6]. Moreover, NHEJ as a major repair pathway in reaction to DSB is error-prone and introduces unpredictable deletions and insertions [30], whereas error-free HDR creates precise sequence changes [7]. The HDR pathway can only occur at the end of the S and G2 phases of the cell cycle [6,7,31]. Thus, the predominance of the first mechanism over the second in plants poses very difficult tasks for researchers in the case of attempts to carry out genomic editing in the knock-in variant. Indeed, in plants, the frequency of knock-out obtained during the restoration of DSB by the NHEJ mechanism is 30–70%, and in some cases up to 100%, while the knock-in frequency is usually at a level of part of a percent or a few percent [7,8].

Our study has shown that rearrangements can occur both with knock-in template carrying flanking DNA homologous to the insertion site in the plant genome and presumably integrated into the genome by the HDR mechanism and with a knock-in template without flanking DNA and presumably integrated into the genome by the NHEJ mechanism. Based on the accounts of flanking genomic DNA sequences following site-specific integration, it can be said that rearrangements at the junction of plant DNA and transgenic construct are rather a normal phenomenon. For example, a maize deletion of approximately 600 bp of the ZMUbi intron of the donor sequence was observed in progeny from two T0 plants, while three T0 plants contained an approximately 400-bp duplication of the right flank homology sequence at the 3′ end of the donor template [18]. Large deletions were also observed in studies on rice when the template for knock-ins was inserted by the NHEJ mechanism [32]. Among the lines studied by us, both deletions and insertions and duplications are also observed. Most of the rearrangements are observed from the right flank, except for line 29, in which 34 bp unknown DNA, 83 bp of sequence located between the coding sequence of *nptII* and its terminator were inserted, immediately after the sequence of the left flanking sequence of plant DNA, and the first 46 nucleotides of the *nptII* promoter was deleted. It should also be noted that this line showed reduced resistance to kanamycin, which is apparently associated with rearrangements in the promoter region of the kanamycin resistance gene (*nptII*). When using a combination of T-DNA and Cas9, rearrangements are also observed on both sides of the integrated construct in work on rice. Overall, the 5′ end of the T-DNAs were relatively well conserved (0–37 bp deletion) compared to the 3′ ends (1–926 bp deletion). The five T-DNAs integrated at the target site in a site –specific manner had larger deletions than the randomly integrated T-DNAs at the left borders: 42–926 bp vs. 1–10 bp [33]. Clarke et al. [34] showed that Cas9 can be efficiently dislodged by RNA polymerase only when a gRNA targets the template strand, indicating that Cas9 has a stronger association to the protospacer side than to the protospacer-adjacent motif (PAM) at the DSB site. It is thus likely that the PAM side of the DSB end is released first and joined with the 5′ end of the integrated template by NHEJ [33].

Perhaps more promising for obtaining knock-ins is the system CRISPR/Cas12a, also known as CRISPR/Cpf1. Cas12a has two different features that seem to be beneficial for GT, compared to Cas9. First, the induced DSB site is close to one end of the protospacer, far away from the PAM sequence. This feature possibly lets the target be cleaved multiple times, thus increasing the chances of GT, without being blocked by mutations induced by NHEJ. Second, Cas12a produces 5′ protruding ends following DSB induction. Utilizing CRISPR/Cas12a in rice also achieved high GT efficiency of up to 8% and all independently identified targeted insertions resulted in precise genomic site integration events, leaving intact junction regions between genomic DNA and upstream homology arm DNA, and downstream homology arm and corresponding genomic DNA [35].

The uneven distribution of rearrangements observed by us on the right and left sides of the side construction can be explained due to a combination of HR and NHEJ or by the fact that in our case, the integration occurred according to the one-sided invasion model of recombination [36], which suggests that homology to only one end of the DSB is sufficient for HDR. One must also to keep in mind that even if a DSB is repaired at one end by an HR mechanism, HR does not need to occur at the other end of the DSB either [31]. Examples have also been reported for site-specific insertions in which one junction was repaired by HR and the other by NHEJ [6].

Unfortunately, we were not able to fully establish the pattern of rearrangements on the right side of plant DNA in four of our five lines. We believe that one reason for difficulties in characterizing this rearrangement is the relatively large size of the affected sequence. In three out of five lines (38, 4-1 and I6), DNA sequences homologous to both vectors used to create knock-ins and lying outside both the T-DNA and the template for knock-ins were identified. However, a detailed analysis of the alignment allows us to assert that in line 38, the inserted additional parts of the plasmid DNA sequence originate from plasmid pIFN (H3.3).3, and not from Cas9H33. In plasmids pIFN (H3.3).2 and pIFN (H3.3).3, knock-in templates are surrounded by Cas9 recognition sites. Such sites are necessary for excising the knock-in template from the plasmid and its linearization. The presence of this sites significantly increases the frequency of knock-ins [15,18,19]. However, in some cases, apparently, the template is not excised from the plasmid or is not completely excised, which leads to the insertion of additional fragments of plasmid DNA into the plant genome. The presence of a homologous DNA region in both plasmids (pIFN (H3.3).2 or pIFN (H3.3).3 and Cas9H33) and an excess of plasmid DNA in a cell created during bio-ballistic transformation can possibly result in rearrangements. Modern concepts of the mechanisms of HDR suggest that repeated sequences in the genome can account for genome rearrangements due to template switching between diverged repeated sequences during DSB repair process [37,38,39]. We have found the insertions of same plasmid DNA in forward and reverse orientation into the plant genome in two lines—150 and 160 bp in line 38 and 170 and 260 bp in line I6. This plasmid sequence is located in pIFN (H3.3).2 and pIFN (H3.3).3 plasmids immediately after the site of excision of the template from plasmid DNA. The fact that rearrangements were detected for both variants of the knock-in template (with and without flanks) indicates that the presence of flanks does not affect the occurrence of mutations.

The high number of DNA rearrangements at the insertion site could potentially be explained by the high level of genetic variability in the suspension cell culture. The level of genetic variability of cultivated plant cells depends on the duration of their cultivation in vitro. Young cell cultures in the first months of cultivation are characterized by an increase in heterogeneity [40,41]. Long-term cultivated cells are usually characterized by genome stabilization accompanied by the sustaining of permanent ploidy level and decrease of the mutation rate [42,43]. This cell line, at the time of our study, has been cultivated in suspension for more than 15 years and, as previously shown, this cell line is characterized by a low level of genetic variability [44]. Accordingly, we assume that the presence of rearrangements at the site of insertion of foreign DNA into a DSB is part of the normal process of DSB reparation in the cell.

Despite the fact that we successfully obtained a series of *A. thaliana* suspension cell lines carrying a site-specific insertion of target genes, none of the lines was without rearrangements in integrated DNA. Apparently, in order to obtain a knock-in with intact plant and plasmid DNA junctions, it is necessary to carefully plan the composition of genetic constructs used to obtain the knock-in, avoiding repetitive elements whenever possible. Finally, the sequences of the flanking regions of loci modified by knock-in should be investigated and reported, to prove that both newly formed junctions arose via HDR at the target locus and are intact.

## 4. Materials and Methods

### 4.1. Plant Material

The fast-growing cell line of *A. thaliana* (L.) Heynh (Columbia ecotype, Col-0 inbred line) was used as the initial plant material. This cell line deposited in the All-Russian collection of higher plant cell cultures (ARCCCHP, http://www.ippras.ru/cfc/alccmp/ (accessed on 16 August 2021)) under No. 85 and designated as NFC-0 was kindly provided by Ph.D. Nosov A.V. (Timiryazev Institute of Plant Physiology RAS, Moscow, Russia) [44]. The cell culture was maintained in vitro on SH medium [45] with the addition of phytohormones (1 mg/L 2,4-D and 0.1 mg/L kinetin).

### 4.2. Plasmids Carrying Cas9 and Guide RNA

The plasmids pBlu/gRNA (no. 59188) for an intermediate cloning step and Cas9 MDC123 (no. 59184) with the Cas9 endonuclease gene under the control of the 2_35S CaMV promoter optimized for expression in *Glycine max* cells were donated by R. Stupar [46] from the Addgene repository. The pBlu/gRNA plasmid carrying an sgRNA cassette under the control of the *A. thaliana* U6 promoter was used as an intermediate vector for inserting selected spacer regions into the sgRNA sequence.

### 4.3. The Sources of Elements of Genetic Constructs for Knock-In

Nucleotide sequences for creating genetic constructs were obtained by PCR using appropriate oligonucleotides and templates. The genomic DNA of *A. thaliana* was used as a template for the amplification of sequences flanking the integration site of the target gene. The signal peptide sequence directing the synthesized protein to the apoplast was amplified on a *Daucus carota* genomic DNA template. The plasmid pGEX4T-1 was the source of the GST (glutathione S-transferase) gene encoding the tag for affinity purification of the target protein. For the amplification of the *nptII* gene sequences (neomycin phosphotransferase II) and the CaMV35S promoter of the cauliflower mosaic virus, the pBi121 plasmid was used as a template. The *dIFN* (deltaferon) gene, a recombinant analog of human interferon gamma for the plant cells expression, was amplified from the pIFN--trp2-D plasmid [47].

### 4.4. Selection of Guide RNA

The region of the histone H3.3 gene HTR5 was chosen as the region for the integration of the target gene. The CRISPOR software (http://crispor.tefor.net, Santa Cruz, CA, USA) was used to select the guide RNA for knock-in into the region of the HTR5 gene. The sequence for knock-in was located between the locus of histone H3.3 gene HTR5 (At4g40040) and the adjacent gene encoding the 12 kDa subunit of microsomal signal peptidase (At4g40042), preceding the coding region of the HTR5 gene.

### 4.5. Creation of Genetic Constructs for Knock-In

The selected sequences that determine the specificity of the guide sgRNA were transferred into the Cas9 MDC123 plasmid using the pBlu/gRNA intermediate plasmid using the corresponding oligonucleotides (Table 1): for the H3.3 histone gene region, 1_gRNA_H Up and 1_gRNA_H Lo.

**Figure 2:**
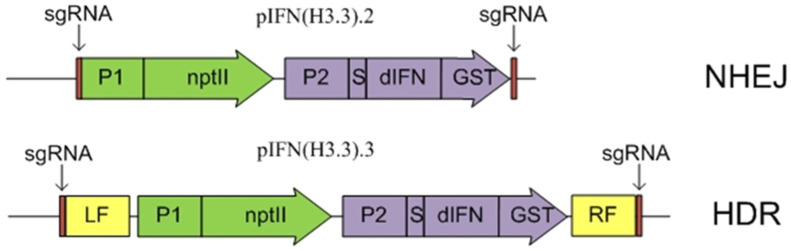
Diagrams of genetic constructs for delivery of the dIFN gene to the region of the histone H3.3 gene. Designations: LF and RF, left and right flanking sequences homologous to the corresponding regions in the *A. thaliana* genome (intergenic region before the H3.3 histone gene); P1-P-NOS promoter of *Agrobacterium tumefaciens* nopaline synthase; P2, CaMV35S promoter of the cauliflower mosaic virus; nptII, neomycin phosphotransferase II gene providing plant cell resistance to kanamycin; S, is the DNA sequence encoding the leader signal of the carrot extensin gene, which ensures the transport of deltaferon into the apoplast; dIFN, DNA sequence encoding the target dIFN protein; GST, DNA sequence encoding a tag for protein affinity purification; sgRNA—Cas9 endonuclease recognition sites identical to the recognition site in the intergenic region upstream of the *A. thaliana* histone H3.3 gene, for excision of the construct from the plasmid in the cell.

For delivery to the H3.3 histone gene region of the target *dIFN* gene (encodes human γ-interferon) and the selective *nptII* gene (provides resistance of transformed cells to kanamycin), two types of genetic constructs pIFN (H3.3).2 and pIFN (H3.3).3 were used, the schemes of which are shown in Figure 2. To increase the frequency of the integration of target genes to the target region, they were flanked by a fragment recognized by Cas9, which ensured their excision from the plasmid in the cell in the form of a linear structure. A detailed description of the creation of this plasmids is described in our previous works [19,24].

### 4.6. Biolistic Transformation of A. thaliana Cells: Obtaining Transgenic Suspension Cultures

Delivery of the target dIFN gene to the region of the HTR5 gene of *A. thaliana* was carried out using biolistic transformation with the immobilization of plasmids pIFN (H3.3).2 or pIFN (H3.3).3 together with the plasmid Cas9H33 on the gold particles. In total, 10 biolistic transformations were performed for each construct.

Callus transformation was performed using a PDS1000/He gene gun (BioRad, Hercules, CA, USA) with the following biolistic parameters: particle size, 0.6 µm; membrane burst pressure, 1100 pci; the vacuum pressure in the chamber was 27 mm Hg, and the distance to the explant was 6 cm. Immobilization of an equimolar mixture of plasmids onto gold particles, one of which included the plasmid with the target gene, and the other with the Cas9H33 plasmid, was carried out according to the method of the gene gun manufacturer. For each transformation, 6 Petri dishes with prepared calli were used, and each callus was fired twice.

Three days after the biolistics procedure, the calli were transferred to selective SH medium of the same composition with kanamycin at a concentration of 100 mg/L and cultivated in light with an intensity of 20 thousand lux at a photoperiod of 18/6 h (day/night). Passages to fresh media of the same composition were carried out weekly. The resulting transgenic, antibiotic-resistant callus was transferred into cell suspensions. Suspensions were cultivated in the dark on an orbital shaker with a stirring intensity of 160 rpm. Subsequently, monoclones were obtained from these cultures by plating the original cell suspensions. To select cell clones, the principle of maximum dilution of cell suspensions of each of the obtained cell lines was used with alternating passages on liquid and agar media. A 4-day-old suspension culture of plant cells was filtered through a sieve with a pore size of 0.9 mm, 1 mL of the filtrate passed through the sieve was obtained, diluted 3–4 times with SH medium, and 1 mL of the diluted filtrate was applied to the surface of a Petri dish with agar medium SH containing 100 mg/L kanamycin. The cells were spread evenly over the surface of the agar with a Drygalski spatula. After 2 weeks, separately grown plant cell clones of each of the lines were passaged onto a fresh SH medium of the same composition. Ten monoclonal calluses were selected for each of the original cell lines; one of these calluses, the most promising in terms of cytological characteristics, was introduced in a suspension culture for further study.

### 4.7. Identification of Cell Lines with Targeted Insertion of Transgenes

DNA was isolated from the calli and PCR analysis was performed using the appropriate primers. Genomic DNA was isolated using CTAB buffer according to the Allen protocol [48]. To confirm the integration of the target gene into the target region of the H3.3 histone gene, PCR was used with primers, one of which was located inside the transferred construct, and the second in the plant DNA region outside the flanking sequence present in the construct (Lo_plan3 and Up_H3.3_1, Table 1). Additionally, as a control, PCR was performed with primers located inside the construct (npt1, npt2, dIFN1 and dIFN2, Table 1).

### 4.8. Detection of Rearrangements at the Target Insertion Site of Transgenes

**Figure 3:**
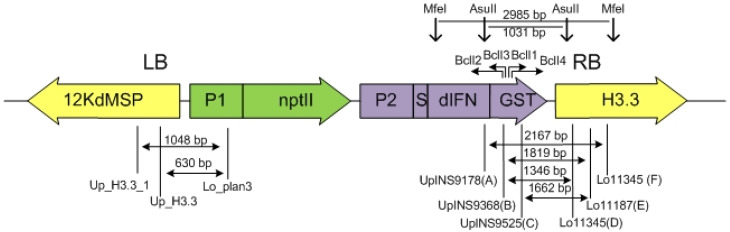
Mutual arrangement of primers and restriction endonucleases used for the analysis of plant and plasmid DNA junctions. Designations: LB and RB, left and right borders of plant DNA; P1-P-NOS promoter of *A. tumefaciens* nopaline synthase; P2, CaMV35S promoter of the cauliflower mosaic virus; nptII, neomycin phosphotransferase II gene providing plant cell resistance to kanamycin; S is the DNA sequence encoding the leader signal of the carrot extensin gene, which ensures the transport of deltaferon into the apoplast; dIFN, DNA sequence encoding the target dIFN protein; Lo_plan3, Up_H3.3, Up_H3.3_1—primers used to identify the left border of the knock-in, the sizes of the resulting fragments are indicated above the arrows in bp; UpINS9178 (A), UpINS9368 (B), UpINS9525 (C), Lo10714 (D), Lo11187 (E), Lo11345 (F)—primers used to identify the right border of the knock-in by direct PCR, the sizes of the resulting fragments are indicated above the arrows in bp; MfeI, AsuII—restriction sites of enzymes used for the inverted PCR method, the size of restriction fragments is indicated in bp; BclI1, BclI2, BclI3, BclI4—primers used for inverted PCR.

The target gene insertion sites in the plant genome were analyzed in two ways. To identify the left border of the insert, PCR was used with primers Lo_plan3 and Up_H3.3 for knock-ins obtained with the construct pIFN (H3.3).2 (without plant flanks in the construct) and Lo_plan3 and Up_H3.3_1 for knock-ins obtained with the construct pIFN (H3.3).3 (carrying flanks homologous to plant DNA) (the location of the primers is shown in Figure 3). The PCR fragments obtained using these primers were subjected to Sanger sequencing (Evrogen, Moscow, Russia).

Two approaches were used to identify the right border of the insertion. The first approach—several sequential PCRs with three pairs of primers—the first PCR with primers UpINS9178 (A) and Lo11345 (F), the second PCR, for which a 100-fold diluted mixture obtained in the first PCR was used, with primers UpINS9368 (B) and Lo11187 (E), or UpINS9525 (C) and Lo11187 (E), or UpINS9368 (B) and Lo10714 (D) (the primer sequence is presented in Table 1, the mutual arrangement of primers is shown in Figure 3). The fragments obtained after the second PCR were excised from the gel, cloned into the pJet1.2 plasmid using the CloneJET PCR Cloning Kit (ThermoFS, Waltham, MA, USA) and subjected to Sanger sequencing (Evrogen, Russia).

The second approach used the inverted PCR method. Total genomic DNA preparations (200 ng) were treated with MfeI or AsuII restriction enzymes, after which the preparations were treated with ligase and, using the resulting mixture as a template, two consecutive PCRs were performed, the first with BclI1 and BclI2 primers, the second with BclI3 and BclI4 primers (the primer sequence is presented in Table 1, primer and restriction site positions are shown in Figure 3). The fragments obtained after the second PCR were excised from the gel, cloned into the pJet1.2 plasmid using the CloneJET PCR Cloning Kit (ThermoFS, USA) and subjected to Sanger sequencing (Evrogen, Russia).

Analysis of the obtained nucleotide sequences was carried out using ClustalW multiple alignment in the Unipro UGENE v37.0 software (Novosibirsk, Russia).

## 5. Conclusions

In this work, we have shown that site-specific insertion of foreign DNA successfully occurs with constructs oriented both to insertion by the mechanism of homologous recombination, carrying flanking regions of DNA homologous to the insertion site, and to insertion by the mechanism of non-homologous end joining, without flanking DNA. At the same time, rearrangements at the junctions of plant and transgenic DNA were detected in the lines obtained with both constructs. Thus, both mechanisms cannot guarantee accurate, error-free site-specific insertion of DNA upon knock-in. The size of the rearrangements can be quite significant up to several hundred nucleotides, and more complex rearrangements were found for the right flank of the insert than for the left. In this case, the rearrangements can consist not only of additional plasmid DNA, but also the elements of the template for knock-in on both sides of the construct can be rearranged. When planning an experiment, this fact must be taken into account, and it may be justified to add additional “ballast” DNA regions at both ends of the knock-in template to protect key structural elements from rearrangements occurring during site-specific integration.

## Figures and Tables

**Table 1:** Oligonucleotides used in the work.

Oligonucleotide	Oligonucleotide Sequence	
1_gRNA_H Up	GATTCGGCCTAAACTAAATCCGAA	Sequence for guide RNA
1_gRNA_H Lo	AAACTTCGGATTTAGTTTAGGCCG	
npt1	CGACGTTGTCACTGAAGCG	nptII marker gene
npt2	AAGCACGAGGAAGCGGTCAG	
dIFN 1	GATGTAGCGGATAATGGAACTCTTTT	dIFN target gene
dIFN 2	TGACTCCTTTTTCGCTTCCCTG	
Up_H3.3	TAGGCAACGATGGTAAAGCGGATT	Testing of the left border
Up_H3.3_1	AATCGCATAATCAAGAAAATCAAAACCC	
Lo_plan3	AGCCGAATAGCCTCTCCACCCAA	
Lo10714 (D)	TCACAATCAAAAGCAATGGCGAGAA	Testing of the right border
Lo11187 (E)	GGTTTCAGTTTCAAGTGGGGAGAGC	
Lo11345 (F)	CGCATCATCATCATCACATTCGCTT	
UpINS9178 (A)	TGACCAGAGCATCCAAAAGAGTGTG	
UpINS9368 (B)	ACAGGGAAGCGAAAAAGGAGTCAGA	
UpINS9525 (C)	GCGATGAAGGTGATAAATGGCGAAA	
BclI1	CAATGTGCCTGGATGCGTTC	
Bcl2	CAACATCAAGAGCGTCATACA	
BclI3	ATAAAACAACATCAAGAGCGTCA	
BclI4	GGCCTTTGCAGGGCTGGCAA	

**Table 2:** Mutations from the left (LF) and right (RF) flanking sequences in the studied cell lines.

Cell Line	Mutations Near LF	Mutations Near RF
**Plasmid pIFN (H3.3).3 with plant flanks**		
29	Intact plant LF, 34 bp unknown DNA, 83 bp of sequence between the coding sequence of *nptII* and T-NOS, P-NOS with truncated first 46 bp *	Parts of P-NOS 98 and 69 bp, deletion of 4 bp of plant RF DNA
38	Intact plant LF, two additional cytosine residues	Part of GST tag from the middle of the gene, 150 and 160 bp from pIFN (H3.3).3 plasmid near RF in forward and reverse orientation interspersed with small parts of undefined plasmid DNA
4-1	Intact plant LF, insertion of 24 bp of *A. thaliana* DNA	150 and 160 bp from pIFN (H3.3).3 plasmid near LF
**Plasmid pIFN (H3.3).2 without plant flanks**		
I1	Intact plant LF, insertion of 17 bp of *A. thaliana* DNA	Deletion of last 15 bp in GST gene, 400 bp from *Cas9* gene from Cas9H33 plasmid
I6	Intact plant LF, two additional cytosine residues	Addition of 2 bp (AC) after GST tag, 170 and 260 bp in forward and reverse orientation from pIFN (H3.3).3 plasmid near RF or from Cas9H33 plasmid

* Mutations are listed in the order in which they appear in the examined DNA (left to right).
